# The Ecology of Collective Behavior

**DOI:** 10.1371/journal.pbio.1001805

**Published:** 2014-03-11

**Authors:** Deborah M. Gordon

**Affiliations:** Department of Biology, Stanford University, Stanford, California, United States of America

## Abstract

Similar patterns of interaction, such as network motifs and feedback loops, are used in many natural collective processes, probably because they have evolved independently under similar pressures. Here I consider how three environmental constraints may shape the evolution of collective behavior: the patchiness of resources, the operating costs of maintaining the interaction network that produces collective behavior, and the threat of rupture of the network. The ants are a large and successful taxon that have evolved in very diverse environments. Examples from ants provide a starting point for examining more generally the fit between the particular pattern of interaction that regulates activity, and the environment in which it functions.

## Abstract 2

Networks of local interactions regulate biological systems. Ecological constraints set by resource distribution, operating costs, and the threat of rupture produce similar collective behavior in ants, cells, and gene transcription.

Collective behavior operates without central control to regulate activity and growth. Systems that operate in this way are ubiquitous in nature. Cells act collectively, for example, as networks of neurons to produce sensations, or as patrolling T-cells that mobilize other immune cells to respond to pathogens. Many animal groups regulate their movement without a leader, such as bird flocks that turn in the sky, or fish schools that swerve to avoid predators. Social insects live in colonies, and simple cues, mostly chemical, regulate how colonies forage, maintain their nests, and reproduce.

Over the past 20 years, across all the fields of biology, attention has turned to deciphering how local interactions produce collective global outcomes (e.g., [1]). We see recurring patterns: a small number of network motifs predominate in gene transcription [2]; similar neural circuits are used in different sensory systems [3]; and feedback loops regulate collective behavior in many interacting groups, such as bacteria, fish, dolphins, and social insects [4]–[8].

It is likely that similar interaction patterns are used in many natural collective processes because they have evolved independently under similar pressures [9]. Such pressures are ecological, a consequence of how the collective behavior acts within, and acts upon, a dynamic environment. But an ecological perspective is missing so far from the study of collective regulation, in molecules, cells, and even in animal groups.

In systems biology and neuroscience, many motifs and circuits have been identified, each a process that uses local interactions to regulate activities such as gene transcription, metabolism, or perception. Showing that patterns exist, for example that the distribution of motifs differs from a random one [10] is a first step; the next will be to show how the patterns have evolved to function in relation to a particular environment. A quantitative description of why a process is effective, or a simulation that selects for that process [2],[11]–[14], helps us to understand how it works. But to understand its evolution we need to know its ecological consequences, what problems it solves in a particular environment, and how it is shaped by, and influences, changing conditions [15].

Outlining hypotheses about the fit between collective behavior and its environment can guide the investigation of collective behavior. For example, we now know enough about physiology that we expect animals that live in hot places to have adaptations for heat exchange. In the same way, we can expect the algorithm that dictates collective organization in particular conditions to be tuned to the constraints of those conditions. With respect to the workings of collective biological systems, we are like the European naturalists of the early 19th century, agog in the Amazon. We are searching for general trends amidst enormous diversity and complexity. A framework for the match between process and environmental conditions can provide predictions that guide the investigation of new systems.

Here I consider three environmental constraints that probably shape the evolution of collective behavior: the patchiness of resources, the operating costs of maintaining the interaction network that produces collective behavior, and the threat of rupture of the network. Other important constraints are not considered here to keep this essay brief.

Ants offer many examples of the match between particular environmental constraints and the regulatory processes used in those conditions. The ants are a hugely successful taxon of more than 12,000 species, found in every terrestrial habitat and using every resource. All ant species live in colonies that operate without any central control, using patterns of interaction to regulate activity [6]. We can see how ant colonies regulate their behavior in response to their environments, and this provides a starting point for examining more generally the fit between a pattern of interaction and the environment in which it functions.

## Patchiness in Space and Time

**Figure 1:**
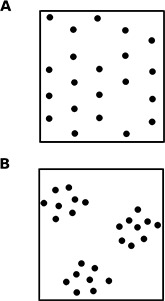
Patchiness in space and time. (A) Uniform. (B) Patchy.

A basic function of collective algorithms is to regulate how the system explores and exploits its environment, searching for and using some resource. How best to search and retrieve depends on the heterogeneity, in space and time, of the resource [16],[17]. Heterogeneity can be characterized by the degree of patchiness (Figure 1). A resource occurs in patches when its presence means there is likely to be more nearby, in space or time. It is not patchy when its occurrence is a Poisson process, so that it pops up in space or time unpredictably.

When resources are uniform in space, the components of a system can engage independently in search and retrieval, without using recruitment. For example, harvester ants in the desert forage for scattered seeds. The seeds are distributed by wind and flooding, and are not patchy in space [18]. An ant can retrieve a seed on its own, and the presence of one seed does not mean that there are likely to be more seeds for others to find. These ants search individually and do not lay pheromone trails to recruit others to seeds.

When resources are patchy in space but not in time, rapid recruitment is useful. In ants this leads to trail formation [19],[20], familiar in many of the ant species that show up in our kitchens. A scouting ant that finds food lays a trail as it returns to the nest, which stimulates other foragers to return to the food, and then lay more chemical trail when they carry the food back to the nest.

Some cellular systems also utilize resources that are patchy in space. In the immune system, T-cells hunt through networks of capillaries for pathogens that are patchy in space, tending to persist in a particular location, and reaching pathogens quickly is helpful because that limits the time available to the pathogens to reproduce and spread. Recruitment by T-cells uses inflammatory signals that activate cells in nearby lymph nodes to respond to the pathogen [21]. Similarly, metastatic cancer cells may use signals from healthy tissue to recruit other cancer cells to a new location [22], if certain areas of tissue constitute an attractive resource, persistent in time and space, for the traveling cells.

When patchy resources persist in both time and space, the system that regulates retrieval can afford the luxury of inflexibility. The red wood ant feeds on the sugary excretions of aphids that suck the sap from trees. A colony forms permanent trails from the nest to the tree, and individual ants travel back and forth on the same trail all their lives. Once an ant becomes associated with a certain foraging direction, it cannot be induced to change trails even for a higher quality resource [23].

Inside cells, bistable signal transduction circuits operate in conditions that are patchy in time, and produce an irreversible transition when an unfavorable condition changes to a favorable one. For example, in frog oogenesis, the MAPK cascade is a bistable signaling system that is triggered by conditions favorable to mating, inducing the production of progesterone that sends oocytes into cell division and on the path to further development [24]. These signaling patterns lead to inflexibility in response to consistent, patchy conditions or resources.

## Operating Costs

**Figure 2:**
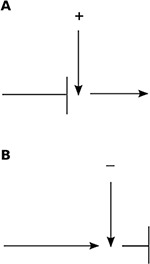
Effect of operating costs. (A) Process that stops unless initiated by a positive event. (B) Process that continues unless stopped by a negative event.

Operating costs create a second set of environmental constraints that influence regulatory processes (Figure 2) [12]. When operating costs are low, the system can keep running unless something stops it, using negative feedback, or repressors: interactions that tend to inhibit or dampen activity. For example, in the humid environment of the tropics, searching costs for ants are low. With so many ants searching every surface, and very high species diversity, ants of different colonies often meet at resources. The probability that an ant stays at a food resource decreases in response to its encounters with ants that do not have the odor of nestmates. Thus ants continue probing every resource unless they are repelled by another colony, and whichever colony has more ants at the resource first is likely to keep it [25].

In gene transcription networks, incoherent loops provide negative feedback that decrease production by repressing transcription once threshold amounts are reached. The system thus bears the cost of producing two products, one of which acts to inhibit the other. As with the tropical ants, low operating costs may make such network motifs more common, here for proteins that are cheaper to produce.

When operating costs are high, so that significant amounts of resources are used merely to keep the interaction network going, regulation works in the opposite direction, to keep activity down except when it is worthwhile. Activity is low unless stimulated by interactions that are likely to occur only when activity is warranted.

For example, harvester ants in the desert lose water by foraging, and get water by metabolizing the fats out of the seeds that foragers collect. Thus the colony must spend water to get water, so operating costs are high. Colonies regulate foraging activity using an autocatalytic process [26]. Outgoing foragers leave the nest in response to interaction with returning foragers with food. Each forager searches until it finds food, so the more food is available, the more quickly foragers find it and the more they stimulate others to leave the nest. When food is scarce there is little foraging and little water loss. Likewise TCP, the protocol that manages traffic congestion in the internet, uses a similar algorithm in which a signal that a data packet has successfully passed a checkpoint stimulates the transmission of further data [27].

Natural selection is currently shaping the collective behavior of harvester ant colonies in response to the high operating cost of foraging in the desert [28]. Variation among colonies in the regulation of foraging activity is associated with reproductive success. Colonies that restrict foraging more in dry conditions, conserving water by using more stringent autocatalysis to stimulate foraging, are the ones likely to have offspring colonies.

In gene transcription networks, activators and repressors act to provide positive and negative feedback [29]. For example, fast-forward loops (FFLs) are based on positive feedback: signal X stimulates Y, which stimulates Z, and Z is not produced unless both X and Y are present. The extra step, in which X stimulates Y, can be regulated when Z is not needed and prevent the production of Z [30],[31]. FFL motifs, based on positive feedback, may be most common in situations when the operating cost of producing Z is especially high.

## Threat of Rupture

The threat of rupture is low when patterns of interaction are protected from interruption. In such environments, permanent connections and specialization can be used. Argentine ants forage on robust, long-lasting trails that connect many nests. Recruitment to new resources is conducted from the permanent trails, not from the nests [32]. Thus the function of the trails, which are rarely ruptured, is specialized in a way similar to our highways and roads; some trails are dedicated to maintaining the flow of ongoing traffic, while others are used for shorter distances and more ephemeral opportunities.

Tissue differentiation in development relies on a low threat of rupture. Cells differentiate in response to interactions with surrounding cells that depend on a slow gradient of signaling pathways across space. For example, in most vertebrate animals brains sit inside hard cases; traumatic injury is rare (and brains are not well adapted to recover from it). Neural functions rely on well-established sets of connections that grow over a long time [33]. In a mammalian brain, experimentally linking retinal projections to the auditory cortex leads auditory neurons to develop the features of and act as visual ones [34], because specialization is produced by the local spatial context, sustained over time. In gene transcription networks, long cascades can be used when the threat of rupture is low enough that there is sufficient time for the many interactions needed to adjust transcription [2].

When the threat of rupture is high, distributed systems and redundancy can be helpful. Distributed systems, in which a given component serves different functions depending on circumstances, are used to create robust large systems in computer engineering. In most ant species studied so far it seems that the allocation of tasks, or functions, to ants, uses a distributed system; changes in conditions shift the rate of local interaction that regulates ant activity [6]. For example, in harvester ants, patrollers change task to become foragers when more food becomes available. In ants distributed systems of task allocation may be a widespread response to rupture due to the frequent loss of ants. The network is more robust if one ant can easily replace another.

**Figure 3:**
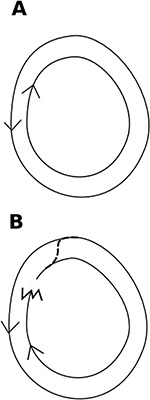
Ring network. (A) Ring network with flow in both directions. (B) When flow in one direction is interrupted, flow in the other direction facilitates recovery.

Redundancy is helpful when the threat of rupture is high because it helps the system to recover quickly. Colonies of the tropical arboreal turtle ant form trails between food resources and a series of nests in the trees [35]. They nest in rotten branches that frequently break and fall down. The ants quickly re-establish the connection between the remaining nests and food sources. A ring network, with signals or ants flowing in both directions, allows for rapid recovery, because after a break in the flow in one direction, the flow in the other direction can re-establish a link (Figure 3). Ring networks are used for similar reasons in fiber optic cable networks, so that one break does not bring down the entire system. In gene transcription networks, dense overlapping regions may tend to occur in regions where rupture or recombination is more likely.

## Conclusion

Ecological constraints—such as heterogeneity in time and space, operating costs, and the threat of rupture—may shape the processes used to regulate activity in many biological systems. Both theoretical and empirical work are needed to investigate this fit, and to move toward a general understanding of the evolution of collective behavior. An ecological perspective can bring together current work in the investigation of diverse complex systems. What an ant does generates, and depends on, the way its colony deals with the world. This is true of many other biological systems; to understand the action of any part, we need to look at what is going on around it.
